# Data for inactivation of free-living *nematode Rhabditida* from water environment using ultraviolet radiation

**DOI:** 10.1016/j.dib.2018.02.074

**Published:** 2018-03-08

**Authors:** Hamed Biglari, Maryam Tatari, Mohammad Reza Narooie, Gholamreza Ebrahimzadeh, Hooshmand Sharafi

**Affiliations:** a Department of Environmental Health Engineering, School of Public Health, Gonabad University of Medical Sciences, Gonabad, Iran; b Department of Biostatistics, Torbat Heydariyeh University of Medical Sciences, Torbat Heydariyeh, Iran; c Department of Environmental Health Engineering, School of Public Health, Iranshahr University of Medical Sciences, Iranshahr, Iran; d Department of Environmental Health Engineering, School of Public Health, Zabol University of Medical Sciences, Zabol, Iran; e Students Research Committee, Kermanshah University of Medical Sciences, Kermanshah, Iran

**Keywords:** Ultraviolet, Water treatment, Nematode, Rhabditida, Disinfection

## Abstract

Sometimes free-living *nematodes* in conventional water treatment processes are not eliminated and cause adverse health effects in water consumer. So, the efficiency of UV lamps (125 W) with irradiation intensity 1020 μW cm^−2^ on inactivation of free-living *nematode Rhabditida* released in water samples has been investigated along with the investigation of the effects of turbidity and change of temperature and exposure time in constant of pH 8 ± 0.2. The results showed that UV radiation could disabled the larval and adult *nematodes* after 12 and 15 min in the presence of turbidity 5 NTU, respectively. Also, increased turbidity up to 50 NTU decreased the inactivation efficiency of larval and adult *nematodes* from 100% to 73% and 64%, respectively. In addition, with increased temperature, the inactivation efficiency increased significantly in a short time. The results showed a significant relationship between increasing exposure time and temperature and turbidity reduction with UV radiation efficiency in the inactivation of the *nematode* (P < 0.00). It was also found that the efficiency of the lamp on *nematode* larvae was more than the adult *nematode*. Therefore, UV radiation can well inactivate larvae and then adult free-living *nematode Rhabditida* in water.

**Specifications Table**

**Table:** 

Subject area	Environmental Sciences
More specific subject area	Biology
Type of data	Tables
How data was acquired	In this descriptive and analytical study, the efficiency of UV lamps (125 W) with irradiation intensity 1020 μW cm^−2^ on inactivation of free-living *nematode Rhabditida* released in water samples has been investigated along with the investigation of the effects of turbidity and change of temperature and exposure time (pH was constant 8 ± 0.2). Free-living *nematode Rhabditida* were prepared and cultured in the laboratory of Gonabad University of Medical Sciences. UV lamp was prepared before each use for 3 to 5 min to warm up and reach a steady state. To move *nematodes* to the sample containers under the process, the plates containing cultured *nematodes* were rinsed by 20 ml of phosphate-buffered saline of pH 8 ± 0.2 so that the *nematodes* were separated from the culture medium and entered into the reactor container.
Data format	Raw, analyzed
Experimental factors	In all experiments, there was control sample and experiments were performed with three replications.
Experimental features	The relationship between inactivation rate and other parameters was evaluated using linear regression test.
Data source location	Gonabad, Mashhad province, Iran
Data accessibility	Data are included in this article

**Value of the data**

• *Nematodes* can also pass live through the bed and enter into the water-supply networks in and endanger human health as carriers of pathogens, therefore, searching for a sure solution to remove them is essential. The results of this study focus on this issue.
• There are various ways to disinfect water resources. Using chlorination has declined because this type of disinfection tends to produce toxic compounds resulting from the use of its components. Therefore, we have to look for other methods of disinfection (for example using ultraviolet radiation). The results of this study emphasize this topic.
• Until now in some studies the effects of UV irradiation, temperature, turbidity to inactivation the different microorganism such as *nematodes* are investigated. But the data from present study specifically studies inactivation of free-living *nematode Rhabditida* from the water using UV radiation was investigated.

## 1 Data

The average chemical quality of water used for growing *nematodes* are shown in Table 1.

**Table 1:** The chemical quality properties of subterranean Gonabad water.

**Total hardness**	**Calcium hardness**	**Hardness of magnesium**	**Ca**	**Na**	**Mg**	**pH**
423.5 ± 19.71	130.95 ± 3.79	292.55 ± 21.31	52.38 ± 1.51	214 ± 16.24	70.21 ± 5.11	7.73

Inactivation efficiency of larval and adult *nematode* up to 15 min of exposure in turbidity 5 NTU is shown in Table 2 (Turbidity 5 NTU, pH 8 ± 0.2). The investigation of results presented in Table 2 showed that with increasing time of exposure to UV lamps, the efficiency of inactivating the larval and adult *nematodes* increased. Larval and adult *nematodes* were inactivated 100% after 12 and 15 min of exposure respectively.

**Table 2:** The inactivation efficiency of *nematodes* at different UV irradiation time.

**Time**[1]	**Larval (%)**	**Adult (%)**
1	4	2
2	16	11
3	23	17
4	32	27
5	47	39
6	55	43
8	72	56
10	89	78
12	100	91
15	100	100

Table 3 shows the inactivation efficiency of *nematodes* in different turbidity at a constant temperature of 20 °C and pH 8 ± 0.2 after a 15 min exposure to UV radiation. Data reported in Table 3 also involve change of control samples. Results of Table 3 show that during the 15 min exposure to UV radiation, increased turbidity up to 15 NTU had no effect on the rate of inactivation of both types of *nematodes* and they were inactivated 100%, but then with increasing turbidity up to 50 NTU, the inactivation efficiency of the larval and adult *nematodes* decreased 73% and 64% respectively. Table 3 also shows that the percentage of the inactivation of larval *nematodes* even in the presence of different levels of turbidity was higher than adult *nematodes*.

**Table 3:** The inactivation efficiency of *nematodes* at different turbidity at a constant.

**Turbidity (NTU)**	**Adult (%)**	**Larval (%)**
5	100	100
6	100	100
7	100	100
8	100	100
9	100	100
10	100	100
15	100	100
20	94	100
25	90	100
30	87	92
40	79	87
50	64	73

Table 4 shows the effect of temperature on the rate of inactivation of *nematodes* during the 2 min exposure time at the presence of turbidity 5 NTU and pH 8 ± 0.2. Results of Table 4 show that increased temperature had increased efficiency of inactivating the *nematodes* and the effect of temperature on inactivation of larval *nematodes* has been more than adult *nematodes* so that at 45 °C, the inactivation of larval and adult *nematodes* was equivalent to 92 and 75%.

**Table 4:** The inactivation efficiency of *nematodes* at different temperature.

**Temperature (°C)**	**Larval (%)**	**Adult (%)**
20	16	11
25	22.4	14.5
30	24.9	18.3
35	49.4	32.8
40	73.2	53.5
45	92	75

## 2 Experimental design, materials and methods

To perform this analytical and descriptive study, Ultraviolet light (254 nm type C Arda Company, Iran- 125 W), flow of 25.3 amps, and intensity of 1020 μW cm^−2^, length of 65 mm, diameter of 10 mm, and arc length of 31 mm was used. The experiment started by placing the lam inside a quartz tube deployed in a 1000 ml glass reactor (mirror to the inside of the container) [2]. The effect of changes in temperature parameters in the range of 20 to 45 °C and turbidity in the range of 5 to 25 NTU within 1 to 15 min on inactivation of larval and adult free-living *nematode Rhabditida* in natural water samples taken from the subterranean Gonabad water (Ghasabe) was investigated. Free-living *nematode Rhabditida* were prepared and cultured in the laboratory of Gonabad University of Medical Sciences. UV lamp was prepared before each use for 3 to 5 min to warm up and reach a steady state. To move *nematodes* to the sample containers under the process, the plates containing cultured *nematodes* were rinsed by 20 ml of phosphate-buffered saline of pH 8 ± 0.2 so that the *nematode*s were separated from the culture medium and entered into the reactor container. In all experiments, there was control sample and experiments were performed with three replications [3], [4]. Except when studying the effect of temperature, using circulating water-cooling system, the water temperature was kept constant on 20° during the test [5]. At the end, the relationship between inactivation rate and other parameters was evaluated using linear regression test by SPSS software (version.21).
