# Supplementary material for: Data for inactivation of free-living nematode Rhabditida from water environment using ultraviolet radiation
Source: Data Brief. 2018 Mar 8;18:30–4. doi: 10.1016/j.dib.2018.02.074 (PMC5995771; doi:10.1016/j.dib.2018.02.074)
Supplement: Supplementary file 1 — Supplementary material [file mmc1.docx]

Dear editor(s)

Data in Brief

Greetings!

At this time, this article with entitled” ***Data for Inactivation of Free-living Nematode Rhabditida from Water Environment Using Ultraviolet Radiation*** “has not been published or submitted in any other journals. We hope that the results of this research will be of interest to you. None of the authors of this paper have any conflicts of interest to declare.

Sincerely yours

Corresponding author

Hooshmand Sharafi
